# Rare gene deletions in genetic generalized and Rolandic epilepsies

**DOI:** 10.1371/journal.pone.0202022

**Published:** 2018-08-27

**Authors:** Kamel Jabbari, Dheeraj R. Bobbili, Dennis Lal, Eva M. Reinthaler, Julian Schubert, Stefan Wolking, Vishal Sinha, Susanne Motameny, Holger Thiele, Amit Kawalia, Janine Altmüller, Mohammad Reza Toliat, Robert Kraaij, Jeroen van Rooij, André G. Uitterlinden, M. Arfan Ikram, EuroEPINOMICS CoGIE Consortium, Federico Zara, Anna-Elina Lehesjoki, Roland Krause, Fritz Zimprich, Thomas Sander, Bernd A. Neubauer, Patrick May, Holger Lerche, Peter Nürnberg

**Affiliations:** 1 Cologne Center for Genomics, University of Cologne, Cologne, Germany; 2 Cologne Biocenter, Institute for Genetics, University of Cologne, Cologne, Germany; 3 Luxembourg Centre for Systems Biomedicine, University of Luxembourg, Esch-sur-Alzette, Luxembourg; 4 Psychiatric and Neurodevelopmental Genetics Unit, Massachusetts General Hospital and Harvard Medical School, Boston, Massachusetts, United States of America; 5 Program in Medical and Population Genetics, Broad Institute of MIT and Harvard, Cambridge, Massachusetts, United States of America; 6 Stanley Center for Psychiatric Research, Broad Institute of MIT and Harvard, Cambridge, Massachusetts, United States of America; 7 Department of Neurology, Medical University of Vienna, Vienna, Austria; 8 Department of Neurology and Epileptology, Hertie Institute for Clinical Brain Research, University of Tübingen, Tübingen, Germany; 9 Institute for Molecular Medicine FIMM, University of Helsinki, Helsinki, Finland; 10 Institute of Human Genetics, University of Cologne, Cologne, Germany; 11 Department of Internal Medicine, Erasmus Medical Center, Rotterdam, the Netherlands; 12 Departments of Epidemiology, Neurology, and Radiology, Erasmus Medical Center, Rotterdam, The Netherlands; 13 Laboratory of Neurogenetics and Neuroscience, Institute G. Gaslini, Genova, Italy; 14 Folkhälsan Institute of Genetics, Folkhälsan Research Center, Helsinki, Finland; 15 Neuroscience Center and Research Programs Unit, Molecular Neurology, University of Helsinki, Helsinki, Finland; 16 Department of Neuropediatrics, Medical Faculty University Giessen, Giessen, Germany; 17 Center for Molecular Medicine Cologne (CMMC), University of Cologne, Cologne, Germany; 18 Cologne Excellence Cluster on Cellular Stress Responses in Aging-Associated Diseases (CECAD), University of Cologne, Cologne, Germany; Seoul National University College of Medicine, REPUBLIC OF KOREA

## Abstract

Genetic Generalized Epilepsy (GGE) and benign epilepsy with centro-temporal spikes or Rolandic Epilepsy (RE) are common forms of genetic epilepsies. Rare copy number variants have been recognized as important risk factors in brain disorders. We performed a systematic survey of rare deletions affecting protein-coding genes derived from exome data of patients with common forms of genetic epilepsies. We analysed exomes from 390 European patients (196 GGE and 194 RE) and 572 population controls to identify low-frequency genic deletions. We found that 75 (32 GGE and 43 RE) patients out of 390, i.e. ~19%, carried rare genic deletions. In particular, large deletions (>400 kb) represent a higher burden in both GGE and RE syndromes as compared to controls. The detected low-frequency deletions (1) share genes with brain-expressed exons that are under negative selection, (2) overlap with known autism and epilepsy-associated candidate genes, (3) are enriched for CNV intolerant genes recorded by the Exome Aggregation Consortium (ExAC) and (4) coincide with likely disruptive *de novo* mutations from the NPdenovo database. Employing several knowledge databases, we discuss the most prominent epilepsy candidate genes and their protein-protein networks for GGE and RE.

## Introduction

Epilepsies are among the most widespread neurological disorders with a lifetime incidence of ~3% [1]. They represent a heterogeneous group of different disease entities that, with regard to aetiology, can be roughly divided in epilepsies with an exogeneous/symptomatic cause and those with a genetic cause. Genetic generalized epilepsies (GGE; formerly idiopathic generalized epilepsies) are the most common genetic epilepsies accounting for 30% of all epilepsies. They comprise syndromes such as juvenile myoclonic epilepsy, childhood absence epilepsy and juvenile absence epilepsy. In general, they tend to take a benign course and show a good response to pharmacotherapy. Among focal genetic epilepsies, benign epilepsy with centro-temporal spikes or Rolandic epilepsy (RE) is the most common form. RE has its onset in childhood or early adolescence and usually tapers off around the age of 15.

High-throughput genomic studies raised the number of epilepsy-associated candidate genes to hundreds; nowadays, frequently mutated ones are included in diagnostic gene panels (for recent reviews see [2,3]. Large consortia initiatives such as Epi4k [4] enrolled 1,500 families, in which two or more affected members displayed epilepsy, as well as 750 individuals, including 264 trios, with epileptic encephalopathies and infantile spasms, Lennox-Gastaut syndrome, polymicrogyria or periventricular heterotopias. In addition to the detection of known and unknown risk factors, the consortium found a significant overlap between the gene network of their epilepsy candidate genes and the gene networks for autism spectrum disorder (ASD) and intellectual disability. Intriguingly, epilepsy is the medical condition most highly associated with genetic autism syndromes [5].

Genomic disorders associated with copy number variations (CNVs) appear to be highly penetrant, occur on different haplotype backgrounds in multiple unrelated individuals and seem to be under strong negative selection [6–8]. A number of chromosomal locations suspected to contribute to epilepsy have been identified [9–11][12,13].

A genome-wide screen for CNVs using array comparative genomic hybridization (aCGH) in patients with neurological abnormalities and epilepsy led to the identification of recurrent microdeletions on 6q22 and 1q22.31 [14]. A deletion on 15q13.3 belongs to the most frequent recurrent microdeletions in epilepsy patients; it is associated with intellectual disability, autism, schizophrenia, and epilepsy [15,16]. The recurrence of some CNVs seems to be triggered by the genome structure, namely by the chromosomal distribution of interspersed repetitive sequences (like Alu transposons) or recently duplicated genome segments (large blocks of sequences >10 kbp with >95% sequence identity that constitute five to six percent of the genome) that give rise to nonallelic homologous recombination [6,17].

CNV screening in large samples showed that 34% of heterozygous deletions affect genes associated with recessive diseases [18]. CNVs are thought to account for a major proportion of human genetic variation and have an important role in genetic susceptibility to common disease, in particular neuropsychiatric disorders [19]. Genome-wide surveys have demonstrated that rare CNVs altering genes in neuro-developmental pathways are implicated in epilepsy, autism spectrum disorder and schizophrenia [3,20].

Considering all types of CNVs across two analysed cohorts, the total burden was not significantly different between subjects with epilepsy and subjects without neurological disease [21]; however, when considering only genomic deletions affecting at least one gene, the burden was significantly higher in patients. Likewise, using Affymetrix SNP 6.0 array data, it has recently been shown that there is an increased burden of rare large deletions in GGE [13]. The drawback of the latter approach is that smaller CNVs cannot be detected. Systematic searches of CNVs in epilepsy cohorts using whole-exome sequencing (WES) data, which provides the advantage to identify smaller deletions along with the larger ones, are still missing.

In the present study, we provide the CNV results of the largest WES epilepsy cohort reported so far. We aimed at (1) identifying the genome-wide burden of large deletions (>400kb), (2) studying the enrichment for deletions of brain-expressed exons, in particular those under negative selection, (3) detecting deletions that overlap with previously defined autism and epilepsy candidate genes, and (4) browsing knowledge databases to help understand the disease aetiology.

## Materials and methods

### Patient cohorts

All patients or their representatives, if participants were under age 18, and included relatives, gave their informed consent to this study. All procedures were in accordance with the Helsinki declaration and approved by the local ethics committees/internal review boards of the participating centers. The leading institution was the Ethics Commission of the University and the University Clinic of Tübingen.

GGE cohort: This cohort included 196 subjects with genetic generalized epilepsy. All subjects were of European descent (Italian 81, German 54, Finnish 22, Dutch 11, British 9, Danish 8, Turkish 6, Swedish 3, French 1, Greek 1). The cohort included 117 female subjects (60%). The GGE-diagnoses were childhood absence epilepsy (CAE; n = 94), juvenile absence epilepsy (JAE; 21), juvenile myoclonic epilepsy (JME; 47), genetic generalized epilepsy with generalized tonic-clonic seizures (EGTCS, 27), early-onset absence epilepsy (EOAE, 4), epilepsy with myoclonic absences (EMA, 1), and unclassified GGE (2). Age of epilepsy onset ranged from 1 year to 38 years with a median of 8 years. The majority of subjects derived from multiplex families with at least 2 affected family members (n = 183), thereof 90 families with 3 or more affected members.

RE cohort: This cohort included 204 unrelated Rolandic patients of European ancestry which were recruited from centers in Austria (n = 107), Germany (n = 84), and Canada (n = 13).

Control cohort: We used 445 females and 283 males (728 in total) from the Rotterdam Study as population control subjects [22]. The same cohort was recently used for the screening of 18 GABA_A_-receptor genes in RE and related syndromes [23].

### Workflow for CNV detection

**Fig 1:**
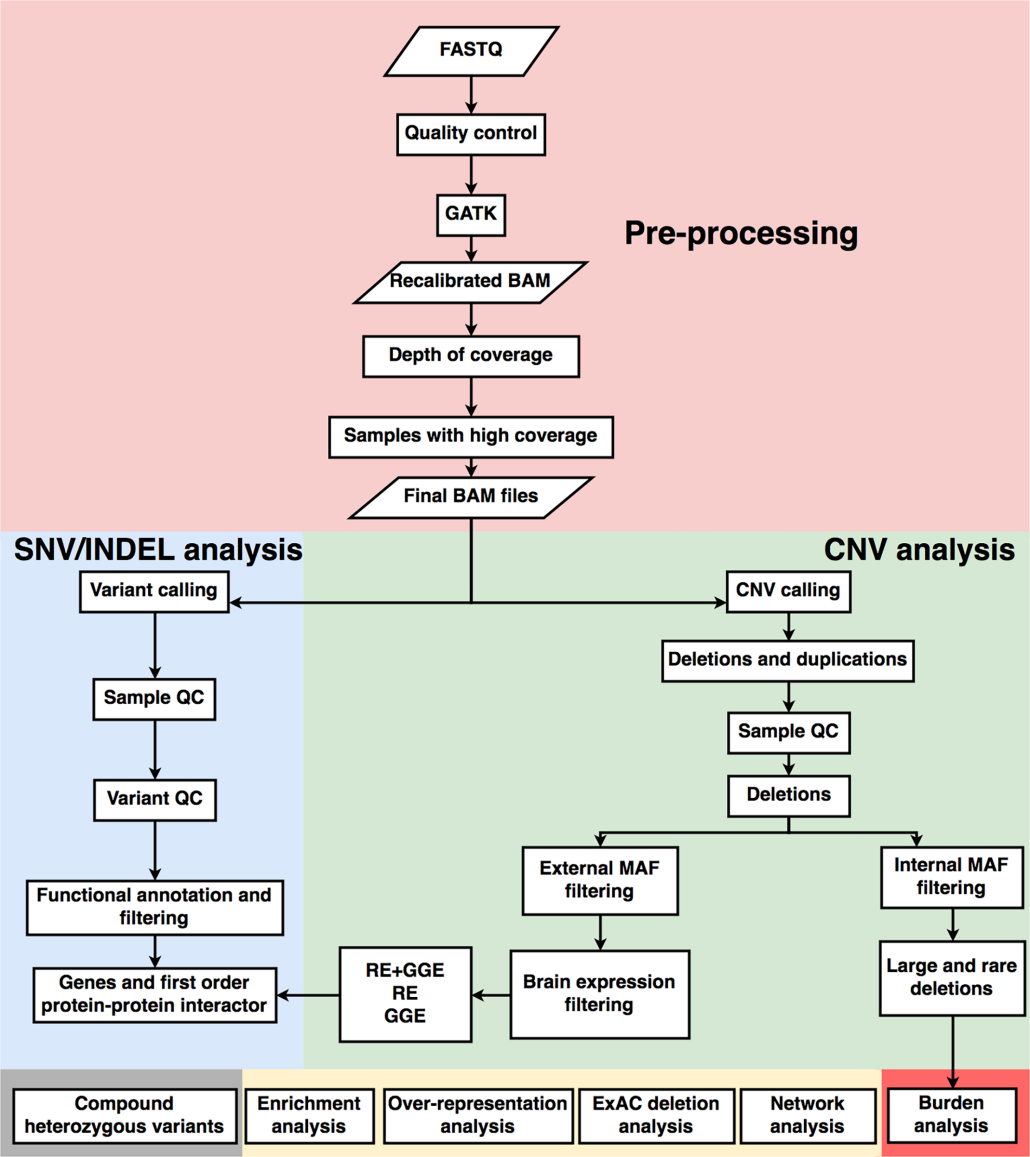
Flowchart of the analysis steps. Parameters used in each step are described in detail in the methods section.

Our primary analysis workflow included three major steps as shown in Fig 1. These are 1) data pre-processing, 2) SNV/INDEL analysis and 3) copy number variant analysis.

Data pre-processing: Sequencing adapters were removed from the FASTQ files with cutadapt [24] and sickle [25]. GATK best practices were followed for the next steps of data pre-processing and variant calling [26]. Alignment to the GRCh37 human reference genome was performed using BWA-MEM [27] with default parameters. Conversion of SAM to BAM files was done with SAMtools [28]. Sorting of BAM files, marking of duplicate reads due to PCR amplification and addition of read group information were done using Picard (https://github.com/broadinstitute/picard) tools with default parameters. Base quality score recalibration and local realignment for INDELs was performed using GATK version 3.2.

Coverage: Mean depth of coverage and target coverage of exons were calculated from the BAM files using the depth of coverage tool from GATK. The same files were also used as input for calling of CNVs.

Variant calling: The GATK haplotype caller (version 3.2) was chosen to perform multiple sample variant calling and genotyping with default parameters. To include splice site variants in the flanking regions of the exons, exonic intervals were extended by 100 bp each upstream and downstream. Multiple sample calling is advantageous in deciding whether a variant can be identified confidently as it provides the genotype for every sample. It allows filtering variants based on the rate of missing genotypes across all samples and also according to the individual genotype.

Sample QC: Samples were excluded from the analysis based on the following criteria: 1) Samples with a mean depth <30x or <70% of exon targets covered at <20x were excluded from further analysis; 2) samples with >3 standard deviations from mean in number of alternate alleles, number of heterozygotes, transition/transversion ratio, number of singletons and call rate as calculated with the PLINK/SEQ i-stats tool (https://atgu.mgh.harvard.edu/plinkseq/); 3) call rate <97%; 4) ethnically unmatched samples as identified by multi-dimensional scaling analysis with PLINK version 1.9 [29]; 5) PI_HAT score>0.25 as calculated by PLINK version 1.9 to exclude related individuals.

Variant QC: Initial filtering of variants was performed based on quality metrics over all the samples with the following parameters for VQSR: Tranches chosen, VQSRTrancheSNV99.90to100.00. QC over all samples (INFO column) was done as follows: a) for SNVs, variants were filtered for QD < 2.0, FS > 60.0, MQ< 40.0, MQRankSum < –12.5, ReadPosRankSum < –8.0, DP <10.0, GQ_MEAN < 20.0, VQSLOD < 0, more than 5% missingness, ABHet > 0.75 or < 0.25 and deviation from Hardy-Weinberg equilibrium (Phred scale p-value of > 20); b) for INDELs, the same was done as for SNVs except for the following parameters for variant filtration: QD <2.0, FS >200.0, ReadPosRankSum < –20.0, DP <10.0, GQ_MEAN <20.0, missingness <5%, Hardy-Weinberg Phred scale value of >20, VQSLOD >0.

To further exclude low quality variants, we also applied filtering based on quality metrics for each genotype using read depth and quality of individual genotypes. Genotypes with a read depth of <10 and GQ of <20 were converted to missing by using BCFtools [28]. Multi-allelic variants were decomposed using variant-tests [30] and left-normalized using BCFtools [28].

Variant annotation: Variants were annotated with ANNOVAR [31] version 2015, Mar22 using RefSeq and Ensembl versions 20150322 and the dbNSFP [32] version 2.6 annotations including nine scores for missense mutations (SIFT, PolyPhen2 HDIV, PolyPhen2 HVAR, LRT, MutationTaster, MutationAssessor, FATHMM, MetaSVM, MetaLR), the CADD score, and three conservation-based scores from GERP++, PhyloP and SiPhy. Splicing variants were defined to include 2 bp before and after the exon boundary position. To obtain rare variants, we filtered the variants for a minor allele frequency (MAF) of <0.005 in public databases such as 1000 genomes [33], dbSNP [34], ExAC (release 0.3) and the exome variant server (EVS). We defined deleterious variants as those variants that fulfil any of the following three criteria: 1) all the variants except the synonymous variants predicted to be deleterious by at least 5 out of 8 missense prediction scores, CADD score >4.5, or 2 out of 3 conservation scores (GERP>3, PhyloP>0.95, SiPHy>10) show high conservation; 2) variants annotated as “splicing”, "stop gain" or "stop loss"; 3) any insertion or deletion.

CNV detection: In the remaining high quality samples, CNVs were detected by using XHMM as described in [35]. In the current study, we focused only on deletions, as the false positive rate for duplications is too high to allow for meaningful interpretation. CNV calls were annotated using bedtools version 2.5 [36]. NCBI RefSeq (hg19, 20150322) was used to identify the genes that lie within the deletion boundaries.

CNV filtering: The detected deletions were filtered based on the following criteria: 1) Z score < –3, given by XHMM; 2) Q_SOME score ≥ 60, given by XHMM.

### Burden analysis of large and rare deletions

Excess deletion rate of the large deletions (length >400 kb) in subjects with epilepsy compared to the controls was measured as described in [13] using PLINK version 1.9 [29]. We set the overlap fraction to 0.7 (70%) and the internal allele frequency cut-off <0.5% and evaluated the significance empirically by 10,000 case-control label permutations.

### Case-only CNVs

The CNVs that are unique for cases (not present in any of the in-house controls) and occur at a low frequency, i.e., present in ≤2 independent cases, while having a frequency of ≤1% in the CNVmap, the DGV gold standard dataset [37] and 1000 genomes SV [38] were selected and subjected to further analysis as described below.

### Validation of CNVs

We proceeded by visual inspection of depth variation across exons of the filtered deletions; we also performed qPCR validations of three small deletions, two of which, *NCAPD2* and *CAPN1*, stood the filtering procedure (see Table 1). For RE patients, genomic DNA samples were analysed using the Illumina OmniExpress Beadchip (Illumina, San Diego, CA, USA) [13]. Twenty-three of 60 CNVs present in the RE patients were validated by available array data (S1 Table). Generally, small CNVs cannot be reliably identified with SNP arrays [39]. Indeed, of the 37 CNVs that were not identified in the beadchip data, 23 have a size of <10 kb, whereas only 2 of the 23 validated CNVs have a size of less than 10 kb according to the array data.

**Table 1:** Epilepsy associated microdeletions.

Type	Chr	Start	End	Z-score	Length	Genes
RE	1	32671406	32673183	-3.78	1777	*IQCC*
RE	1	43296070	43317484	-6.52	21414	*ERMAP*, *ZNF691*
RE	1	53320120	53329849	-3.94	9729	*ZYG11A*
RE	1	55586222	55591447	-4.46	5225	*USP24*
RE	1	115137047	115168530	-3.13	31483	*DENND2C*
RE	1	150252003	150259252	-3.3	7249	*C1orf54*, *CIART*
RE	1	153658548	153662047	-3.44	3499	*NPR1*
RE	1	160061571	160064997	-4.46	3426	*IGSF8*
RE	1	249144392	249212591	-3.25	68199	*PGBD2*, *ZNF692*
RE	2	44502637	44539912	-4.48	37275	*SLC3A1*
RE	3	4403776	4562816	-3.79	159040	*ITPR1*, *ITPR1-AS1*, *SUMF1*
RE	4	169362457	169393930	-3.01	31473	*DDX60L*
RE	5	71519462	71533975	-5.36	14513	*MRPS27*
RE	5	75858199	75914495	-3.32	56296	*F2RL2*, *IQGAP2*
RE	5	96506883	96518935	-4.44	12052	*RIOK2*
RE	5	118965402	118970803	-4.73	5401	*FAM170A*
RE	5	140482462	140531165	-3.11	48703	*PCDHB3*, *PCDHB4*, *PCDHB5*, *PCDHB6*
RE	6	31777772	31779777	-3.97	2005	*HSPA1L*
RE	6	33693196	33703280	-6.64	10084	*IP6K3*
RE	6	44143759	44151705	-3.58	7946	*CAPN11*
RE	6	116441989	116442904	-5.26	915	*COL10A1*, *NT5DC1*
RE	7	74197233	74212576	-3.34	15343	*GTF2IRD2*, *NCF1*
RE	7	100146395	100153393	-4.98	6998	*AGFG2*
RE	8	82571539	82752251	-3.13	180712	*CHMP4C*, *IMPA1*, *SLC10A5*, *SNX16*, *ZFAND1*
RE	8	146028239	146033207	-4.62	4968	*ZNF517*
RE	9	35800178	35801935	-4.58	1757	*NPR2*
RE	9	140243513	140250835	-3.24	7322	*EXD3*
RE	10	5920045	5926074	-5.17	6029	*ANKRD16*
RE	10	49383834	49420140	-4.44	36306	*FRMPD2*
RE	11	2549103	2606577	-3.38	57474	*KCNQ1*
RE	11	4903092	4929495	-4.46	26403	*OR51A7*, *OR51T1*
RE	11	7727796	7818510	-3.74	90714	*OR5P2*, *OVCH2*
RE	11	17533429	17546119	-3.17	12690	*USH1C*
RE	11	47600393	47608426	-5.11	8033	*FAM180B*, *KBTBD4*, *NDUFS3*
RE	11	59189760	59211596	-3.31	21836	*OR5A1*, *OR5A2*
RE	11	64977256	64981526	-6.72	4270	*CAPN1*, *SLC22A20*
RE	12	6625993	6627159	-5.01	1166	*NCAPD2*
RE	12	130922883	130927235	-3.85	4352	*RIMBP2*
RE	14	54863694	55907289	-3.32	1043595	*ATG14*, *CDKN3*, *CGRRF1*, *CNIH1*, *DLGAP5*, *FBXO34*, *GCH1*, *GMFB*, *LGALS3*, *MAPK1IP1L*, *MIR4308*, *SAMD4A*, *SOCS4*, *TBPL2*, *WDHD1*
RE	14	77302503	77327178	-3.27	24675	*LRRC74A*
RE	14	92900208	92920437	-4.01	20229	*SLC24A4*
RE	15	23811123	28525396	-4.21	4714273	*ATP10A*, *GABRA5*, *GABRB3*, *GABRG3*, *GABRG3-AS1*, *HERC2*, *IPW*, *LINC00929*, *LOC100128714*, *MAGEL2*, *MIR4715*, *MKRN3*, *NDN*, *NPAP1*, *OCA2*, *PWAR1*, *PWAR4*, *PWAR5*, *PWARSN*, *PWRN1*, *PWRN2*, *PWRN3*, *PWRN4*, *SNORD107*, *SNORD108*, *SNORD109A*, *SNORD109B*, *SNORD115-1*, *SNORD115-10*, *SNORD115-11*, *SNORD115-12*, *SNORD115-13*
RE	15	29346087	32460550	-4.57	3114463	*APBA2*, *ARHGAP11B*, *FAM7A*, *NA7*, *DKFZP434L187*, *FAM189A1*, *FAN1*, *GOLGA8H*, *GOLGA8J*, *GOLGA8R*, *GOLGA8T*, *HERC2P10*, *KLF13*, *LOC100288637*, *LOC283710*, *MIR211*, *MTMR10*, *NDNL2*, *OTUD7A*, *TJP1*, *TRPM1*, *ULK4P1*, *ULK4P2*, *ULK4P3*
RE	16	9856958	10032248	-5.26	175290	*GRIN2A*
RE	16	70560498	70573138	-4.22	12640	*SF3B3*, *SNORD111*, *SNORD111B*
RE	17	7010272	7017572	-3.43	7300	*ASGR2*
RE	17	10403892	10632442	-3.09	228550	*ADPRM*, *MAGOH2P*, *MYH1*, *MYH2*, *MYH3*, *MYHAS*, *SCO1*, *TMEM220*
RE	17	38346658	38350074	-4.63	3416	*MIR6867*, *RAPGEFL1*
RE	17	73623470	73661285	-4.39	37815	*RECQL5*, *SMIM5*, *SMIM6*
RE	17	76967650	76970921	-4.45	3271	*LGALS3BP*
RE	18	30873076	30928981	-3.86	55905	*CCDC178*
RE	19	9014087	9054377	-4.25	40290	*MUC16*
RE	19	14673265	14677779	-4.49	4514	*NDUFB7*, *TECR*
RE	19	14854191	14884892	-3.24	30701	*ADGRE2*
RE	19	35862216	35941102	-3.7	78886	*FFAR2*, *LINC01531*
RE	19	45447959	45465365	-5.89	17406	*APOC2*, *APOC4*, *APOC4-APOC2*, *CLPTM1*
RE	19	51175236	51192575	-3.33	17339	*SHANK1*
RE	19	52271871	52327971	-3.68	56100	*FPR2*, *FPR3*
RE	20	39830726	39831937	-3.5	1211	*ZHX3*
RE	20	44806537	44845668	-4.27	39131	*CDH22*
RE	20	54823759	54824900	-8.62	1141	*MC3R*
GGE	1	76779478	77094515	-4.34	315037	*ST6GALNAC3*
GGE	1	169510234	169511641	-4.49	1407	*F5*
GGE	2	166852481	166872273	-3.02	19792	*LOC102724058*, *SCN1A*
GGE	3	10331397	10335915	-4.04	4518	*GHRL*, *GHRLOS*
GGE	5	21751815	21854929	-6.45	103114	*CDH12*
GGE	6	43320067	43323250	-3.68	3183	*ZNF318*
GGE	7	13971097	14028735	-5.88	57638	*ETV1*
GGE	7	121651161	121652685	-3.43	1524	*PTPRZ1*
GGE	7	146471346	146829615	-4.41	358269	*CNTNAP2*, *LOC101928700*
GGE	7	150501839	150558285	-3.04	56446	*AOC1*, *TMEM176A*
GGE	8	2944572	3045513	-6.06	100941	*CSMD1*
GGE	8	144391574	144400286	-4.33	8712	*TOP1MT*
GGE	9	21350268	21409671	-3.7	59403	*IFNA13*, *IFNA2*, *IFNA6*, *IFNA8*
GGE	9	97080895	97090973	-5.74	10078	*NUTM2F*
GGE	9	113189903	113550109	-7.57	360206	*MUSK*, *SVEP1*
GGE	10	20432177	20506529	-3.39	74352	*PLXDC2*
GGE	10	55568487	55582740	-4.38	14253	*PCDH15*
GGE	10	90524124	90534348	-5.32	10224	*LIPN*
GGE	10	116919814	117026498	-3.09	106684	*ATRNL1*
GGE	11	26568916	26587286	-3.41	18370	*ANO3*, *MUC15*
GGE	11	40136003	40137868	-3.76	1865	*LRRC4C*
GGE	11	60531165	60621186	-4.85	90021	*CCDC86*, *MS4A10*, *MS4A15*, *PTGDR2*
GGE	11	72465895	72794788	-3.31	328893	*ATG16L2*, *FCHSD2*, *MIR4459*, *MIR4692*, *STARD10*
GGE	11	124844950	124858018	-5.07	13068	*CCDC15*
GGE	12	40749853	41463875	-4.27	714022	*CNTN1*, *LRRK2*, *MUC19*
GGE	12	53073535	53086673	-3.42	13138	*KRT1*, *KRT77*
GGE	12	56825208	56827994	-5.15	2786	*TIMELESS*
GGE	12	91445072	91450028	-3.85	4956	*KERA*
GGE	13	23777841	24895857	-3.6	1118016	*ANKRD20A19P*, *C1QTNF9*, *C1QTNF9B*, *C1QTNF9B-AS1*, *LINC00327*, *MIPEP*, *MIR2276*, *SACS*, *SACS-AS1*, *SGCG*, *SPATA13*, *SPATA13-AS1*, *TNFRSF19*
GGE	16	20471400	20498025	-7.68	26625	*ACSM2A*
GGE	16	56659681	56693111	-4.59	33430	*MT1A*, *MT1B*, *MT1DP*, *MT1E*, *MT1F*, *MT1JP*, *MT1M*
GGE	16	61747707	61859108	-3.57	111401	*CDH8*
GGE	16	89804176	89849549	-4.26	45373	*FANCA*, *ZNF276*
GGE	17	36453091	36485777	-3.98	32686	*GPR179*, *MRPL45*
GGE	17	62850638	62856934	-4.31	6296	*LRRC37A3*
GGE	18	43496355	43604681	-3.89	108326	*EPG5*, *PSTPIP2*
GGE	19	1056280	1061916	-3.36	5636	*ABCA7*
GGE	19	9270761	9272102	-4.04	1341	*ZNF317*
GGE	19	37309563	37619956	-3.24	310393	*ZNF345*, *ZNF420*, *ZNF568*, *ZNF790*, *ZNF790-AS1*, *ZNF829*
GGE	19	58386121	58420835	-3.08	34714	*ZNF417*, *ZNF814*
GGE	20	2463808	2465032	-3.21	1224	*ZNF343*
GGE	20	22562576	23016658	-3.9	454082	*FOXA2*, *LINC01384*, *SSTR4*
GGE	20	58440579	58444005	-3.3	3426	*SYCP2*

### Compound heterozygous mutations and protein-protein interactions

We checked for concurrence of a deletion in one allele and a deleterious variant in the second allele. We included the first order interacting partners from the protein-protein interaction network (PPIN) in this analysis [40] and assessed if any gene or its first order interacting partner carries a deletion in one allele and a deleterious variant in the other. We excluded all genes that had no HGNC (HUGO Gene Nomenclature Committee) entry resulting in a network of 13,364 genes and 140,902 interactions. This network was then further filtered for interactions likely to occur in brain tissues using a curated data set of brain-expressed genes [41]. The final brain-specific PPIN consisted of 10,469 genes and 114,533 interactions.

### Gene-set enrichment analysis

Genes that were expressed in brain [42] and located within deletion boundaries were used as input for an enrichment analysis using the Ingenuity Pathway Analyser (IPA®) [43]. We performed the enrichment analysis with all deleted genes from the RE and GGE samples together as well as for each phenotype separately.

### Over-representation analysis

To assess whether the deleted set of genes were enriched in known epilepsy-associated genes, we retrieved genes that were associated with the disease term “epilepsy” from the DisGeNET database [44]. Then we compared the overlap between the brain-expressed genes that are deleted in RE (n = 85), GGE (n = 49) and RE+GGE (n = 134) against the brain-expressed epilepsy-related genes in DisGeNet (n = 674). We used the total number of brain-expressed genes (n = 14,177) as the background. The R GeneOverlap package (https://bioconductor.org/packages/release/bioc/html/GeneOverlap.html) was used to compute the p-value.

### CNV tolerance score analysis

The CNV tolerance score was used as defined in [45]. The CNV tolerance and deletion scores for the genes that are deleted in our study were obtained from the ExAC database [46] and their enrichment in GGE and RE cases was assessed by the Wilcoxon rank sum test.

### Overlap with different databases

The overlap between the different data sets was obtained by gene symbol matches between the detected gene deletions and the gene lists from different databases; more details are given in the discussion section. A workflow depicting the steps above is shown in Fig 1.

## Results

After quality control, exomes of 390 epilepsy cases (196 GGE, 194 RE) and 572 controls were used for downstream analyses (Fig 1). The final RE and GGE datasets comprised 26,476 and 30,207 variants, respectively.

### Epilepsy-associated microdeletions

75 out of 390 epilepsy patients (~19%) carried a total of 104 case-only deletions spanning 260 genes (see Table 1), which covered a wide size range between 915 bp and 3.11 Mbp. 43 out of 194 RE patients carried deletions compared to 32 out of 196 patients with GGE, thus, we did not observe any significant difference in the total number of deletions between the two disease entities (p-value = 0.68). In the combined dataset, 35 out of 73 were large multigene deletions. Among them were several recurrent deletions (see Table 1), including those located on 15q13.3 and 16p11.2 that were previously reported to be associated with epilepsy and other brain disorders.

### Comparative analysis of Rolandic and GGE candidate genes

Because our cohort is composed of GGE and RE patients, we sought to compare the functional differences between the two subtypes of epilepsies by studying the pathways and functions that are enriched in the respective deleted genes (see Table 2). Initially we performed GO term enrichment without applying any additional filter to the deletion calls and noticed that synaptic and receptor functions are more prominent in RE cases (data not shown). If the deletion calls were filtered for brain-specific gene expression, we observed that, separately and together, GGE and RE-deleted genes are enriched for the functional terms “nervous system development and function”, “behavior” and “tissue morphology”; this functional convergence might have been expected when selecting for brain-expressed genes.

**Table 2:** Physiological system development and function.

Name	p-value
**GGE+RE**	
Nervous System Development and Function	2.74E-02–3.36E-06
Tissue Morphology	2.62E-02–4.20E-06
Behavior Auditory and Vestibular System Development and Function	2.37E-02–3.63E-05
Organ Morphology	2.43E-02–5.29E-04
**RE**	
Nervous System Development and Function	4.90E-02–3.89E-05
Tissue Morphology	4.90E-02–1.34E-04
Behavior	4.90E-02–2.56E-04
Auditory and Vestibular System Development and Function	4.53E-02–2.59E-04
Organ Morphology and Vestibular System Development and Function	4.90E-02–2.59E-04
**GGE**	
Nervous System Development and Function	4.91E-02–2.28E-04
Tissue Morphology	4.07E-02–2.28E-04
Behavior	4.47E-02–4.62E-04
Hematological System Development and Function	3.81E-02–6.79E-04

**Fig 2:**
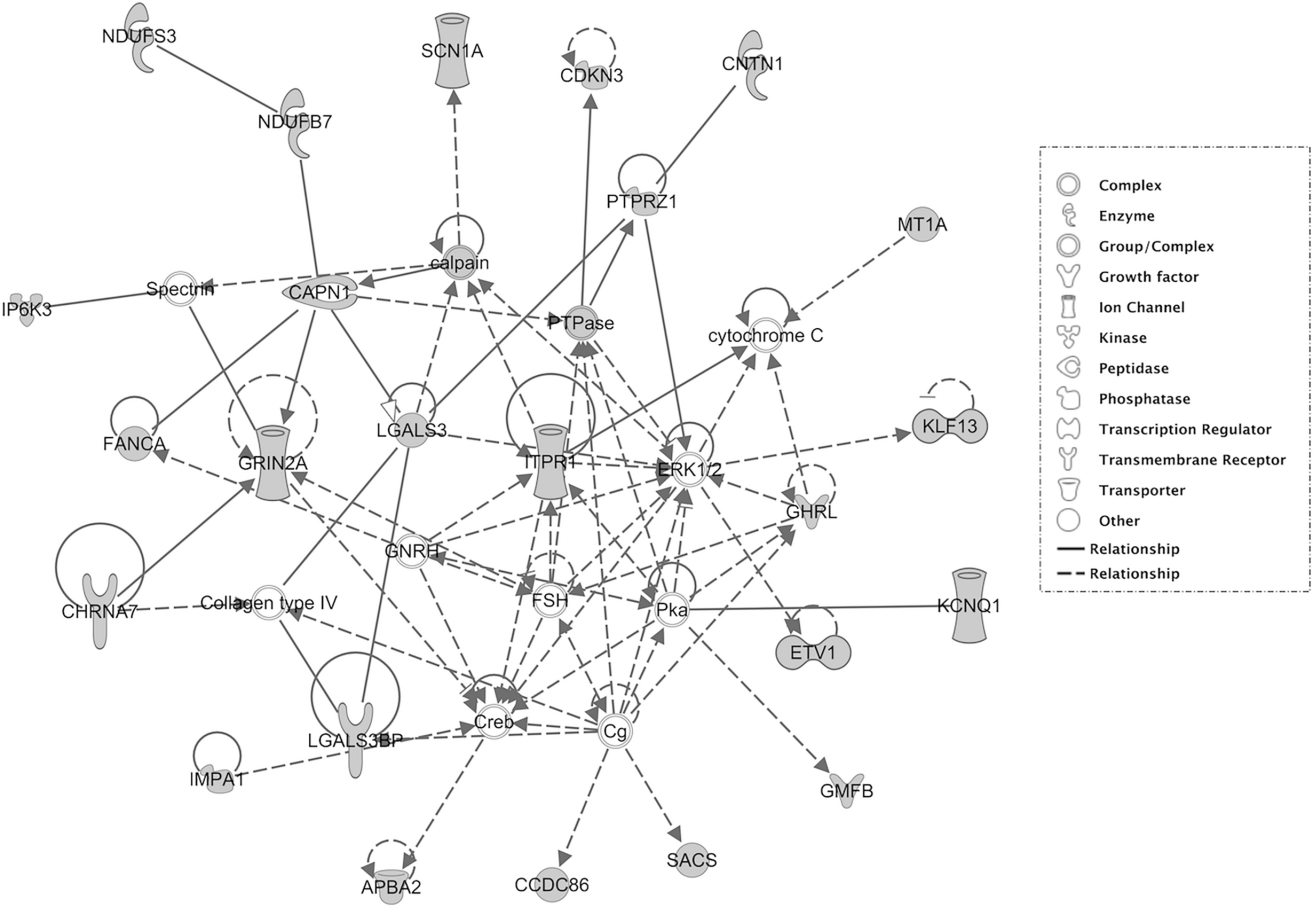
Network analysis of brain-expressed genes filtered by the CNVs identified in both GGE and RE together. The top network from the pathway analysis generated by Ingenuity Pathway Analyser (IPA^**®**^) is shown.

When analysing GGE and RE datasets separately, the top PPIN enriched in GGE is associated with “carbohydrate metabolism”, “small molecule biochemistry” and “cell signaling”, whereas the top network enriched in RE is associated with “neurological disease”, “organismal injury and abnormalities” and “psychological disorders” (see Table 3). The top enriched network including GGE and RE-deleted genes (Fig 2) is described below.

**Table 3:** Top networks.

Rank	Associated network functions
**GGE+RE**	
1	Nervous System Development and Function, Neurological Disease, Behavior
2	Connective Tissue Disorders, Developmental Disorder, Skeletal and Muscular Disorders
3	Cell-To-Cell Signalling and Interaction, Molecular Transport, Small Molecule Biochemistry
4	Cancer, Organismal Injury and Abnormalities, Reproductive System Disease
5	Carbohydrate Metabolism, Lipid Metabolism, Small Molecule Biochemistry
**RE**	
1	Neurological Disease, Organismal Injury and Abnormalities, Psychological Disorders
2	Cell Morphology, Nervous System Development and Function, Tissue Morphology
3	Cellular Development, Cellular Growth and Proliferation, Hematological System Development and Function
4	Embryonic Development, Organismal Development, Tissue Morphology
5	Cellular Compromise, Cell Cycle, Amino Acid Metabolism
**GGE**	
1	Carbohydrate Metabolism, Small Molecule Biochemistry, Cell Signaling
2	Cancer, Organismal Injury and Abnormalities, Endocrine System Disorders
3	Cancer, Dermatological Diseases and Conditions, Organismal Injury and Abnormalities
4	Lymphoid Tissue Structure and Development, Tissue Morphology, Behavior

### Deletion burden analysis

We performed 10,000 case-control label permutations to test whether there is an increased burden of large and rare deletions in cases as compared to the controls (Table 4). We noticed that (1) the deletion rate per individual with at least one deletion in cases compared to the controls showed statistical significance in both GGE and RE (p-value = 1e-04, p-value = 0.011) and (2), considering cumulative length of all large and small deletions, no significant difference between cases and controls was observed in both GGE and RE (p-value = 0.16, p-value = 0.41), indicating that there is no difference in the length of CNVs in cases and controls.

**Table 4:** Burden test showing empirical p-values of cases/controls permutation statistics.

Dataset	Deletion rate per person	Proportion of samples with at least one deletion	Total length of deletions	Average length of deletions
GGE + RE	1.0E-04	1.0E-04	2.7E-01	2.8E-01
GGE	1.0E-04	1.0E-04	1.7E-01	1.8E-01
RE	1.1E-02	3.0E-03	4.1E-01	2.3E-01

### Enrichment for known epilepsy and autism-associated genes

To check the overlap between the deletions detected in our study and genes known to be associated with epilepsy, we searched for overlap with the genes listed (n = 499) in the EpilepsyGenes database [47]. This led to the following set of 8 genes: *CHRFAM7A*, *CHRNA7*, *SCN1A*, *CNTNAP2*, *GABRB3*, *GRIN2A*, *IGSF8*, *ITPR1*. *The GRIN2A* deletion is from the same patient published earlier [48] and which we used as one of the positive controls in our primary CNV detection pipeline [49]. One should notice that genes such as *CHRNA7* and *GABRB3* are located within larger deletions containing other genes; so they might be questionable as *bona fide* epilepsy-associated genes.

Using the core autism candidate genes (n = 455 genes) present in *brainspan* [50], we identified 13 deleted genes: *APBA2*, *ATP10A*, *CDH22*, *CDH8*, *GABRA5*, *GABRG3*, *NDN*, *NDNL2*, *CNTNAP2*, *GABRB3*, *GRIN2A*, *SCN1A and SHANK1* (Table 5). This set is particularly enriched in GO terms “neuron parts” and “transporter complexes”. Note that *GABRB3* and *GABRG3* belong to multigenic large deletions (Table 1).

**Table 5:** Overlap with specific gene sets.

PSD genes	BCG genes	Autism brainSpan	EpilepsyDB	clinVar
*NDUFS3*	*APBA2*	*APBA2*	*CHRFAM7A*	*SACS*
*RIMBP2*	*ATRNL1*	*ATP10A*	*CHRNA7*	*CNTNAP2*
*TJP1*	*CDH22*	*CDH22*	*SCN1A*	*GABRB3*
*CNTN1*	*CSMD1*	*CDH8*	*CNTNAP2*	*GRIN2A*
*CNTNAP2*	*ETV1*	*GABRA5*	*GABRB3*	*ITPR1*
*GABRB3*	*FAN1*	*GABRG3*	*GRIN2A*	*SCN1A*
*GRIN2A*	*GMFB*	*NDN*	*IGSF8*	
*HSPA1L*	*IGSF8*	*NDNL2*	*ITPR1*	
*IGSF8*	*NPR2*	*CNTNAP2*		
*PTPRZ1*	*OTUD7A*	*GABRB3*		
*SHANK1*	*PLXDC2*	*GRIN2A*		
	*SCN1A*	*SCN1A*		
	*ZFAND1*	*SHANK1*		
	*ZNF343*			
	*ZNF568*			
	*CNTN1*			
	*CNTNAP2*			
	*GABRB3*			
	*GRIN2A*			
	*ITPR1*			
	*PTPRZ1*			
	*SHANK1*			

PSD (postsynaptic density); BCG (Brain Critical Genes). Genes common to at least two of the compared sets are highlighted in grey.

### Deletions of brain-critical exons

Disorders such as autism, schizophrenia, mental retardation and epilepsy impact fecundity and put negative selection pressure on risk alleles. In a recent report [7] exome and transcriptome data from large human population samples were combined to define a class of brain-expressed exons that are under purifying selection. These exons that are highly expressed in brain tissues and characterized by a low mutation burden in population controls were called “brain-critical exons” (n = 3,955); the associated genes were accordingly called “brain-critical genes” (BCG, n = 1,863) [3].

Twenty-two deleted genes are in common with the BCG set (see Table 5). The *SHANK1* deletion is found in a single RE case. It spans 17,339 bp (8 exons out of 9). There is only one report on the possible implication of the deletion of this gene in childhood epilepsy [51]. A deletion of *ITPR1* is observed in another RE case; this deletion affects also *SUMF1*, but this gene was filtered out by the BCG overlap selection. The deletion of *CNTN1* in a GGE patient encompasses in addition *MUC19* and *LRRK2*, the latter is a known Parkinson candidate gene [52].

### Exome Aggregation Consortium deletions

The ExAC data comprise 60,706 unrelated individuals sequenced as part of various disease-specific and population genetic studies. Deletions annotated in ExAC (release 0.3.1 of 23/08/16) were identified, similar to the present study, by read depth analysis using XHMM [45]. We sought to compare those CNV calls with the ones detected in the present work. Out of the 260 deleted genes detected in our study, 164 genes (67%) showed deletions in ExAC too (see S2 Table). Several genes highlighted in the previous paragraphs were ranked high using the CNV tolerance score defined by [45]. However, we did not identify a significant difference, neither in CNV tolerance scores (p-value = 0.53) nor in CNV deletion scores (p-value = 0.22), between GGE and RE-deleted genes. This may indicate that GGE and RE deletions are equally likely to fall into the same category of ExAC deletion calls.

### Compound heterozygous and first order protein-protein interaction mutations

Compound heterozygous mutations play a role in many disease aetiologies such as autism and Parkinson’s disease [53–55]. We searched for possibly deleterious non-synonymous changes in the parental undeleted gene copy, but we did not detect any hemizygous variant that had a critical intolerance score (see Methods). Subsequently, we hypothesised that simultaneous mutations in proteins which interact directly (first-order protein interactors) may increase the associated deleterious effect. Within a curated brain-specific PPIN (see Methods, [40]), we inspected first order interacting proteins with potentially deleterious mutations or exon losses (see Table 6) and found a few interesting hits, including *SPTAN1* that interacts directly with *SHANK1*; *SPTAN1* encodes alpha-II spectrin and is known to be associated with epilepsy [56,57]. A remarkable and unique case of multiple hits was observed in a patient who accumulated four hits: the originally detected *ITPR1* deletion and three potentially deleterious non-synonymous SNVs in *RYR2*, *HOMER2* and *STARD13*. *RYR2* (ryanodine receptor 2) and *ITPR1* (inositol-1,4,5-trisphosphate receptor 1) have been independently reported to be implicated in brain disorders. *RYR2 de novo* mutations have been identified in patients with intellectual disability [58] and activation of *ITPR1* and *RYR2* can lead to the release of Ca^2+^ from intracellular stores affecting propagating Ca^2+^ waves [59]. *HOMER2*, a brain-expressed gene, has been reported to be involved in signalling defects in neuropsychiatric disorders [60]. The *STARD13* locus has been reported to be associated with aneurysm and sporadic brain arteriovenous malformations [61,62].

**Table 6:** First order protein-protein interaction hits.

Gene with deleterious SNV/INDEL	Gene within deletion boundaries	Type	CHR	position	ref	alt	annotation
*LACTB*	*MRPS27*	RE	15	63421767	T	C	exonic
*SPEN*	*SF3B3*, *SNORD111*, *SNORD111B*	RE	1	16254645	G	A	exonic
*NRG1*	*SF3B3*, *SNORD111*, *SNORD111B*	RE	8	32406278	A	G	exonic
*SPTAN1*	*SHANK1*	RE	9	131367308	T	G	splicing
*STARD13*	*ITPR1*, *ITPR1-AS1*, *SUMF1*	RE	13	33700223	C	T	exonic
*RYR2*	*ITPR1*, *ITPR1-AS1*, *SUMF1*	RE	1	237730032	A	G	exonic
*HOMER2*	*ITPR1*, *ITPR1-AS1*, *SUMF1*	RE	15	83561556	G	C	exonic
*EPS15L1*	*AGFG2*	RE	19	16528403	C	T	exonic
*DDX41*	*U2SURP*	GGE	5	176939650	G	C	splicing

### Over-representation of gene-disease associations

DisGeNET is a discovery platform integrating information on gene-disease associations from public data sources and literature [44]. The current version (DisGeNET v4.0) contains 429,036 associations between 17,381 genes and 15,093 diseases ranked according to supporting evidence. Over-representation analysis of genes that are deleted in both GGE and RE together (134 genes) showed significant over-representation (empirical p-value = 0.012) of epilepsy-associated genes (*APBA2*, *CHRNA7*, *CNTNAP2*, *F5*, *GABRA5*, *GABRB3*, *GRIN2A*, *KCNQ1*, *MT1E*, *PTPRZ1*, *SCN1A*, *SGCG*, *SSTR4*). We observed a similar result for GGE (49 genes; empirical p-value = 0.009; overlapping genes: *CNTNAP2*, *F5*, *MT1E*, *PTPRZ1*, *SCN1A*, *SGCG*, and *SSTR4*), but we did not see an over-representation in RE (85 genes; empirical p-value = 0.217; overlapping epilepsy genes are *APBA2*, *CHRNA7*, *GABRA5*, *GABRB3*, *GRIN2A*, and *KCNQ1*). This may reflect the heterogeneous risk factors in adulthood epilepsies compared to RE.

### Protein-protein interaction network analysis

We searched for network modules carrying a higher deletion burden with Ingenuity Pathway Analyser (IPA^**®**^). Considering GGE and RE together and using brain-expressed genes as an input for IPA we identified a total of 12 networks. The identified network scores ranged from two to 49 and the number of focus molecules in each network ranged from one to 24. Of all the 12 identified networks, the network shown in Fig 2 is the top-ranked network with a score of 49 and 24 focus molecules. It is associated to the terms “Nervous system development and function”, “Neurological disease” and “Behavior” (see Table 3). The network reveals an interesting module where the genes *CAPN1*, *GRIN2A*, *ITPR1*, *SCNA1* and *CHRNA7* are central. Interestingly, *CAPN1* is well ranked (no deletion or duplication) in the ExAC CNV records (S2 Table) and it is not covered by BCG, epilepsy and autism data sets used in this study.

### Enrichment for likely disruptive *de novo* mutations

Many studies on neuropsychiatric disorders such as autism spectrum disorder, epileptic encephalopathy, intellectual disability and schizophrenia have utilized massive trio-based whole-exome sequencing (WES) and whole-genome sequencing (WGS). Epilepsy candidate genes with *de novo* mutations (DNMs) were searched in the NeuroPsychiatric De Novo Database, NPdenovo [63]. DNMs were found in *GABRB3*, *SHANK1*, *ITPR1*, *GRIN2A*, *SCN1A*, *PCDHB4* and *IQGAP2*.

## Discussion

We analysed a WES dataset of 390 epilepsy patients (196 GEE, 194RE) for microdeletions. The deletion rate per individual with at least one deletion in cases compared to 572 controls showed statistical significance in both GGE and RE. Enrichment for known epilepsy and autism genes led to gene sets with synaptic and receptor functions which were mainly represented in Rolandic cases. The top PPIN enriched in GGE was associated with “carbohydrate metabolism”, “small molecule biochemistry” and “cell signaling”, whereas the top networks associated with RE are “neurological disease”, “organismal injury and abnormalities” and “psychological disorders”, this is reminiscent of our previous attempt to classify metabolic and developmental epilepsies [3].

Among single-gene deletions, *CDH22*, *CDH12* and *CDH8* are of particular interest; *CDH12* is a cadherin expressed specifically in the brain and its temporal pattern of expression seems to be consistent with a role during a critical period of neuronal development [64]. Moreover, a group of cadherins, *CDH7*, *CDH12*, *CDH18* and *PCDH12*, are reported to be associated with bipolar disease and schizophrenia [65]. The smallest deletion (1,166 bp) that we could detect in this study concerns *NCAPD2*; this gene is annotated in the autismkb database [66]. It is an important component of the chromatin-condensing complex, which is highly conserved across metazoan. This gene was previously found to be associated with Parkinson's disease [39] and its paralog *NCAPD3* is associated with developmental delay [67].

Deletions of brain-critical exons pointed to the *ITPR1* deletion, which has been reported to be associated with spinocerebellar ataxia type 16 [68,69]. *CNTN1* is another deletion of interest, the gene is highly expressed in fetal brain, it encodes a neural membrane protein which functions as a cell adhesion molecule and may be involved in forming axonal connections/growth and in neuronal migration in the developing nervous system [70,71]. Moreover, its paralogs *CNTN2* and *CNTN4* are associated with epilepsy [72] and autism [73], respectively. Interestingly, in the ExAC data, the brain-expressed genes *ITPR1* and *CNTN1* show the third and fourth highest intolerance score ranks, respectively (S2 Table).

Protein-Protein interaction network analysis revealed the *CAPN1* deletion as an interesting candidate gene; this is a double gene loss (4,270 bp) spanning *CAPN1* (exon 17 to 22 out of 22 exons) and *SLC22A1* (exon 1 out of 10 exons). *SLC22A1*, a transporter of organic ions across cell membranes, is lowly expressed in the brain, whereas *CAPN1* is highly expressed in the brain. Calpain1 (*CAPN1*) belongs to the calcium-dependent proteases, which play critical roles in both physiological and pathological conditions in the central nervous system. They are also recognized for their synaptic and extra-synaptic neurotoxicity and neuro-protection [74]. Several ion channels, including *GRIN2A* [75] are calpain substrates. Further, a missense mutation in *CAPN1* is associated with spino-cerebellar ataxia in the Parson Russell terrier dog breed [76] and has recently been reported in humans with cerebellar ataxia and limb spasticity [77].

Additional candidate genes can be identified on the periphery of the IPA network (see Fig 2): 1) *CNTN1* (commented on above), 2) *SACS*, for which a large deletion (> 1Mb) was found, and 3) the single gene deletion of *KCNQ1* (~ 57 kb). For *SACS*, a SNV is reported to be associated with spastic ataxia [78] and epilepsy [79]. *KCNQ1* and its paralog *KCNQ3* are subunits forming an expressed neuronal voltage-gated potassium channel. Further, hypomorphic mutations in either *KCNQ2*, an established epilepsy-associated gene [80], or *KCNQ3* are reported to be highly penetrant [81]. *KCNQ1* is co-expressed in heart and brain; it is found in forebrain neuronal networks and brainstem nuclei, regions in which a defect in the ability of neurons to repolarize after an action potential can produce seizures and dysregulate autonomic control of the mouse heart [82], yet one should be cautious as no validation is available for human.

Enrichment for likely disruptive *de novo* mutations in several genes suggests that deletions of these genes could cause a similar phenotype as in the NPdenovo and consequently will be penetrant in the heterozygotic state. This is indeed the case for *ITPR1*, for which recessive and dominant *de novo* mutations causing Gillespie syndrome [83], a rare variant form of aniridia characterized by non-progressive cerebellar ataxia, intellectual disability and iris hypoplasia, have been described. Two of the genes, which we have identified as *ITPR1* interactors, *RYR2* and *SPTAN1*, are also DNM genes in DPdenovo.

In summary, by filtering and comparison to genes that are (1) evolutionary constrained in the brain, (2) implicated in autism and epilepsy, (3) spanned by ExAC deletions, or (4) affected by neuropsychiatric associated *de novo* mutations, we observed a significant enrichment of deletions in genes potentially involved in neuropsychiatric diseases, namely *GRIN2A*, *GABRB3*, *SHANK1*, *ITPR1*, *CNTN1*, *SCN1A*, *PCDHB4*, *IQGAP2*, *SACS*, *KCNQ1* and *CAPN1*. Interaction network analysis identified a hub connecting many of the epilepsy candidate genes identified in this and previous studies. The extended search for likely deleterious mutations in the first order protein-protein interactions and NPdenovo database pointed to the potential importance of *ITPR1* deletion alone or in combination with *RYR2* and *SPTAN1* deleterious mutations.

We are aware that the set of epilepsy exomes that we screened for CNVs in the present study, although the largest analyzed so far, is still small given the genetic complexity of the disease and its population frequency. However, this study appears to provide a contrasting view to the genetic bases of childhood and juvenile epilepsies, as the top protein–protein interactions showing that GGE deleted proteins are preferentially associated with metabolic pathways, whereas in RE cases the association is biased towards neurological processes. Scrutinizing of additional patients’ exomes/genomes and transcriptomes should provide an efficient way to understand the disease aetiology and the biological processes underlying it. The results presented here may contribute to the understanding of epilepsy genetics and provide a resource for future validations to improve diagnostics.

## Supporting information

S1 Table Deletions present in array data. (DOCX)

S2 Table Deletions in common with ExAC CNVs. Data is sorted from low to high deletion score (del.score) and duplication (dup) frequencies. "+" indicates expression in the brain. Deletion score increases with increasing intolerance.
(DOCX)
