# Supplementary material for: Rare gene deletions in genetic generalized and Rolandic epilepsies
Source: PLoS One. 2018 Aug 27;13(8):e0202022. doi: 10.1371/journal.pone.0202022 (PMC6110470; doi:10.1371/journal.pone.0202022)
Supplement: S1 Table — (DOCX) [file pone.0202022.s001.docx]

**S1 Table. Deletions present in array data.**

| **Chr** | **Start** | **End** | **Length** | **Genes** | **Present in regions to filter** |
| --- | --- | --- | --- | --- | --- |
| 1 | 43296070 | 43317484 | 21414 | *ERMAP, ZNF691* | No |
| 1 | 53320120 | 53329849 | 9729 | *ZYG11A* | Yes |
| 1 | 115137047 | 115168530 | 31483 | *DENND2C* | No |
| 2 | 44502637 | 44539912 | 37275 | *SLC3A1* | No |
| 3 | 4403776 | 4562816 | 159040 | *ITPR1, ITPR1-AS1, SUMF1* | No |
| 4 | 169362457 | 169393930 | 31473 | *DDX60L* | No |
| 5 | 71519462 | 71533975 | 14513 | *MRPS27* | No |
| 5 | 75858199 | 75914495 | 56296 | *F2RL2, IQGAP2* | No |
| 5 | 96506883 | 96518935 | 12052 | *RIOK2* | Yes |
| 5 | 140482462 | 140531165 | 48703 | *PCDHB3, PCDHB4, PCDHB5, PCDHB6* | Yes |
| 6 | 33693196 | 33703280 | 10084 | *IP6K3* | No |
| 8 | 82571539 | 82752251 | 180712 | *CHMP4C, IMPA1, SLC10A5, SNX16, ZFAND1* | No |
| 10 | 49383834 | 49420140 | 36306 | *FRMPD2* | No |
| 14 | 54863694 | 55907289 | 1043595 | *ATG14, CDKN3, CGRRF1, CNIH1, DLGAP5, FBXO34, GCH1, GMFB, LGALS3, MAPK1IP1L, MIR4308, SAMD4A, SOCS4, TBPL2, WDHD1* | No |
| 14 | 77302503 | 77327178 | 24675 | *LRRC74A* | No |
| 15 | 29346087 | 32460550 | 3114463 | *APBA2, ARHGAP11B, CHRFAM7A, CHRNA7, DKFZP434L187, FAM189A1, FAN1, GOLGA8H, GOLGA8J, GOLGA8R, GOLGA8T, HERC2P10, KLF13, LOC100288637, LOC283710, MIR211, MTMR10, NDNL2, OTUD7A, TJP1, TRPM1, ULK4P1, ULK4P2, ULK4P3* | No |
| 15 | 23811123 | 28525396 | 4714273 | *ATP10A, GABRA5, GABRB3, GABRG3, GABRG3-AS1, HERC2, IPW, LINC00929, LOC100128714, MAGEL2, MIR4715, MKRN3, NDN, NPAP1, OCA2, PWAR1, PWAR4, PWAR5, PWARSN, PWRN1, PWRN2, PWRN3, PWRN4, SNORD107, SNORD108, SNORD109A, SNORD109B, SNORD115-1, SNORD115-10, SNORD115-11, SNORD115-12, SNORD115-13, S* | No |
| 16 | 9856958 | 10032248 | 175290 | *GRIN2A* | No |
| 17 | 10403892 | 10632442 | 228550 | *ADPRM, MAGOH2P, MYH1, MYH2, MYH3, MYHAS, SCO1, TMEM220* | No |
| 17 | 73623470 | 73661285 | 37815 | *RECQL5, SMIM5, SMIM6* | Yes |
| 18 | 30873076 | 30928981 | 55905 | *CCDC178* | Yes |
| 19 | 45447959 | 45465365 | 17406 | *APOC2, APOC4, APOC4-APOC2, CLPTM1* | No |
| 20 | 54823759 | 54824900 | 1141 | *MC3R* | No |
