# Supplementary material for: Rare gene deletions in genetic generalized and Rolandic epilepsies
Source: PLoS One. 2018 Aug 27;13(8):e0202022. doi: 10.1371/journal.pone.0202022 (PMC6110470; doi:10.1371/journal.pone.0202022)
Supplement: S2 Table — Data is sorted from low to high deletion score (del.score) and duplication (dup) frequencies. "+" indicates expression in the brain. Deletion score increases with increasing intolerance. (DOCX) [file pone.0202022.s002.docx]

**S2 Table. Deletions in common with ExAC CNVs.**

| **HGNC** | **Brain** | **CHR** | **START** | **END** | **cds_len** | **gene_len** | **del** | **dup** | **del.score** | **dup.score** | **cnv.score** |
| --- | --- | --- | --- | --- | --- | --- | --- | --- | --- | --- | --- |
| IQCC | + | 1 | 32671236 | 32674288 | 1669 | 3052 | 41 | 1 | -2.623544152 | 0.583195479 | -2.471627899 |
| ERMAP | + | 1 | 43282795 | 43310660 | 1551 | 27865 | 1 | 8 | 0.480748455 | -0.823379015 | -0.425215558 |
| ZNF691 | + | 1 | 43312280 | 43318148 | 1096 | 5868 | 0 | 0 | 0.022331951 | 0.493769336 | 0.503231742 |
| USP24 | + | 1 | 55532032 | 55680786 | 7740 | 148754 | 0 | 2 | 2.042565836 | 1.53282633 | 1.884072981 |
| ST6GALNAC3 | + | 1 | 76540404 | 77100286 | 987 | 559882 | 0 | 1 | 0.635401501 | 0.481903843 | 0.71332483 |
| C1orf54 | + | 1 | 150240600 | 150253327 | 546 | 12727 | 3 | 12 | 0.124516266 | -1.045379554 | -0.641860823 |
| IGSF8 | + | 1 | 160061130 | 160068733 | 1808 | 7603 | 0 | 1 | 0.287817859 | 0.301696075 | 0.423784119 |
| F5 | + | 1 | 169483404 | 169555826 | 6838 | 72422 | 4 | 1 | -0.449909425 | 1.137009467 | 0.5336849 |
| ZNF692 | + | 1 | 249144205 | 249153343 | 1540 | 9138 | 6 | 24 | -1.868951238 | -2.531252393 | -2.471627899 |
| SLC3A1 | + | 2 | 44502599 | 44548633 | 2365 | 46034 | 23 | 45 | -2.623544152 | -2.531252393 | -2.471627899 |
| SCN1A | + | 2 | 166845670 | 166984523 | 6186 | 138853 | 9 | 5 | -1.810908478 | 0.043445991 | -0.670522876 |
| SUMF1 | + | 3 | 3742498 | 4508965 | 1179 | 766467 | 4 | 9 | -0.513018336 | -0.908525108 | -0.764074106 |
| ITPR1 | + | 3 | 4535032 | 4889524 | 8519 | 354492 | 0 | 6 | 1.725139512 | 0.253387244 | 0.753565178 |
| GHRL | + | 3 | 10327359 | 10334631 | 483 | 7272 | 1 | 0 | 0.372479739 | 1.214689608 | 1.048091047 |
| MRPS27 | + | 5 | 71515236 | 71616473 | 1642 | 101237 | 2 | 1 | -0.277487506 | 0.63115712 | 0.340595652 |
| IQGAP2 | + | 5 | 75699074 | 76003957 | 5978 | 304883 | 32 | 13 | -2.623544152 | -1.141733115 | -2.435588281 |
| RIOK2 | + | 5 | 96496571 | 96518964 | 1978 | 22393 | 6 | 18 | -1.509592757 | -2.256647924 | -1.976393386 |
| PCDHB3 | + | 5 | 140480234 | 140483406 | 2397 | 3172 | 1 | 5 | 1.657469966 | 0.899130927 | 0.99912774 |
| PCDHB4 | + | 5 | 140501581 | 140505201 | 2394 | 3620 | 40 | 3 | -2.623544152 | 2.201582277 | -0.988534575 |
| PCDHB5 | + | 5 | 140514800 | 140517703 | 2394 | 2903 | 41 | 4 | -2.623544152 | 2.02513866 | -0.646985259 |
| PCDHB6 | + | 5 | 140529683 | 140532868 | 2391 | 3185 | 5 | 2 | 1.667827598 | 2.38494211 | 1.914791919 |
| HSPA1L | + | 6 | 31777396 | 31783437 | 1932 | 6041 | 0 | 0 | 0.401632891 | 0.531737845 | 0.661692239 |
| IP6K3 | + | 6 | 33689444 | 33714762 | 1263 | 25318 | 0 | 0 | 0.335329745 | 0.724944238 | 0.801391166 |
| ZNF318 | + | 6 | 43274872 | 43337216 | 6798 | 62344 | 1 | 0 | 0.119892425 | 0.965453183 | 0.79947501 |
| NT5DC1 | + | 6 | 116422012 | 116570660 | 1605 | 148648 | 4 | 3 | -1.027729486 | 0.024943674 | -0.400335605 |
| COL10A1 | + | 6 | 116440086 | 116479910 | 2055 | 39824 | 3 | 1 | -0.75927544 | 0.440689832 | -0.066585525 |
| ETV1 | + | 7 | 13930853 | 14031050 | 1624 | 100197 | 3 | 6 | -0.672793719 | -0.645347304 | -0.682951535 |
| AGFG2 | + | 7 | 100136834 | 100165842 | 1291 | 29008 | 2 | 3 | 0.6545631 | 0.895985923 | 0.849625612 |
| PTPRZ1 | + | 7 | 121513143 | 121702090 | 7128 | 188947 | 8 | 2 | -1.568023817 | 0.841591482 | -0.198054466 |
| CNTNAP2 | + | 7 | 145813453 | 148118090 | 4037 | 2304637 | 5 | 6 | 0.563947177 | 0.204322538 | 0.472669463 |
| TMEM176A | + | 7 | 150497491 | 150502208 | 751 | 4717 | 2 | 0 | -0.169683976 | 1.154291398 | 0.700844273 |
| CSMD1 | + | 8 | 2792875 | 4852494 | 10919 | 2059619 | 7 | 30 | 0.106786859 | -1.998685059 | -1.148652918 |
| IMPA1 | + | 8 | 82570196 | 82598928 | 1061 | 28732 | 2 | 2 | -0.062286695 | 0.494818479 | 0.324510263 |
| ZFAND1 | + | 8 | 82613569 | 82645138 | 1037 | 31569 | 3 | 3 | -0.713229432 | -0.029152124 | -0.288960927 |
| SNX16 | + | 8 | 82711816 | 82755101 | 1077 | 43285 | 1 | 2 | 0.456422625 | 0.505492858 | 0.576511245 |
| TOP1MT | + | 8 | 144386554 | 144442149 | 2288 | 55595 | 22 | 8 | -2.623544152 | -0.919375543 | -2.360916245 |
| ZNF517 | + | 8 | 146024261 | 146036554 | 1529 | 12293 | 81 | 4 | -2.623544152 | -0.583788753 | -2.471627899 |
| NPR2 | + | 9 | 35792151 | 35809729 | 3356 | 17578 | 19 | 13 | -2.623544152 | -1.04407072 | -1.716095807 |
| SVEP1 | + | 9 | 113127531 | 113342160 | 11061 | 214629 | 2 | 6 | 0.548008965 | 0.157194253 | 0.329345173 |
| EXD3 | + | 9 | 140201348 | 140317714 | 2707 | 116366 | 0 | 13 | 0.684156271 | -1.725317858 | -1.203058829 |
| ANKRD16 | + | 10 | 5903580 | 5931869 | 1128 | 28289 | 2 | 3 | -0.532263044 | -0.149995699 | -0.304101007 |
| PLXDC2 | + | 10 | 20105168 | 20578785 | 1674 | 473617 | 2 | 1 | -0.086459784 | 0.734142595 | 0.487533876 |
| ATRNL1 | + | 10 | 116853124 | 117708503 | 4386 | 855379 | 3 | 5 | 0.10741737 | 0.044128336 | 0.111085788 |
| KCNQ1 | + | 11 | 2465914 | 2870339 | 2168 | 404425 | 3 | 3 | -0.518480243 | 0.154714284 | -0.106873975 |
| USH1C | + | 11 | 17515442 | 17565963 | 2942 | 50521 | 6 | 1 | -1.083452944 | 1.162495114 | 0.188844589 |
| LRRC4C | + | 11 | 40135753 | 41481323 | 1929 | 1345570 | 0 | 0 | 0.710652242 | 0.483380747 | 0.758829916 |
| NDUFS3 | + | 11 | 47586888 | 47606114 | 1663 | 19226 | 1 | 0 | 0.695145694 | 1.400181735 | 1.327943672 |
| KBTBD4 | + | 11 | 47599277 | 47599823 | 677 | 546 | 0 | 0 | 0.477900568 | 0.734800622 | 0.865962201 |
| CCDC86 | + | 11 | 60609544 | 60618554 | 1107 | 9010 | 11 | 2 | -2.623544152 | 0.070262779 | -1.498783415 |
| CAPN1 | + | 11 | 64948037 | 64979477 | 2230 | 31440 | 0 | 0 | 0.821376542 | 1.16710151 | 1.305530475 |
| STARD10 | + | 11 | 72465774 | 72504726 | 853 | 38952 | 2 | 2 | -0.614402312 | 0.070403966 | -0.194948524 |
| ATG16L2 | + | 11 | 72525353 | 72554719 | 2355 | 29366 | 0 | 4 | 1.005027614 | 0.048613563 | 0.425402526 |
| FCHSD2 | + | 11 | 72547790 | 72853306 | 2271 | 305516 | 2 | 4 | -0.198729335 | -0.160404265 | -0.177850106 |
| NCAPD2 | + | 12 | 6602522 | 6641121 | 4392 | 38599 | 8 | 4 | -1.341991076 | 0.505722435 | -0.21514961 |
| LRRK2 | + | 12 | 40590546 | 40763087 | 8008 | 172541 | 11 | 19 | -1.750477101 | -1.477247598 | -1.376714468 |
| CNTN1 | + | 12 | 41086244 | 41466220 | 3278 | 379976 | 0 | 1 | 1.728174309 | 1.425101777 | 1.802168696 |
| TIMELESS | + | 12 | 56810903 | 56843187 | 3665 | 32284 | 0 | 4 | 1.522050316 | 0.446777062 | 0.919722383 |
| KERA | + | 12 | 91444268 | 91451760 | 1071 | 7492 | 0 | 2 | 0.446874734 | -0.000114033 | 0.250670659 |
| RIMBP2 | + | 12 | 130880682 | 131200826 | 3261 | 320144 | 1 | 7 | 0.349561229 | -0.727894178 | -0.409028887 |
| SGCG | + | 13 | 23755091 | 23899304 | 918 | 144213 | 7 | 23 | -0.867575065 | -1.730521173 | -1.415064654 |
| SACS | + | 13 | 23902965 | 24007841 | 2135 | 104876 | 8 | 23 | -1.203293261 | -1.815881184 | -1.563224422 |
| TNFRSF19 | + | 13 | 24144509 | 24250232 | 1335 | 105723 | 12 | 26 | -2.042544874 | -1.987046267 | -1.865746884 |
| CDKN3 | + | 14 | 54863567 | 54886936 | 672 | 23369 | 1 | 0 | 0.123183899 | 0.934097692 | 0.787426186 |
| CNIH1 | + | 14 | 54893654 | 54908149 | 381 | 14495 | 0 | 0 | 0.494550985 | 0.806566767 | 0.947001865 |
| GMFB | + | 14 | 54941202 | 54955914 | 508 | 14712 | 0 | 0 | 0.526211632 | 0.841351244 | 0.980023489 |
| CGRRF1 | + | 14 | 54976530 | 55005567 | 1039 | 29037 | 0 | 2 | 0.565795517 | 0.150343318 | 0.414022933 |
| SAMD4A | + | 14 | 55033815 | 55260033 | 2366 | 226218 | 1 | 0 | 0.549832768 | 1.285731173 | 1.193637347 |
| GCH1 | + | 14 | 55308726 | 55369570 | 892 | 60844 | 0 | 1 | 0.509578001 | 0.480372822 | 0.661531792 |
| WDHD1 | + | 14 | 55405668 | 55493823 | 3540 | 88155 | 3 | 4 | -0.075497723 | 0.242811576 | 0.174122536 |
| SOCS4 | + | 14 | 55493948 | 55516206 | 1329 | 22258 | 0 | 1 | 0.392612172 | 0.162471626 | 0.368963914 |
| MAPK1IP1L | + | 14 | 55518349 | 55536910 | 922 | 18561 | 0 | 0 | 0.211003259 | 0.614292596 | 0.680394542 |
| LGALS3 | + | 14 | 55590828 | 55612126 | 869 | 21298 | 2 | 1 | -0.386319462 | 0.528835801 | 0.228501941 |
| DLGAP5 | + | 14 | 55614830 | 55658396 | 2649 | 43566 | 2 | 56 | 0.02444499 | -2.531252393 | -2.471627899 |
| FBXO34 | + | 14 | 55738021 | 55828636 | 2142 | 90615 | 0 | 2 | 0.175325399 | -0.364821598 | -0.160408258 |
| ATG14 | + | 14 | 55833110 | 55878576 | 1539 | 45466 | 0 | 4 | 0.551818028 | -0.357672317 | -0.034249132 |
| SLC24A4 | + | 14 | 92788925 | 92962596 | 1990 | 173671 | 3 | 7 | -0.48267106 | -0.681136634 | -0.630949501 |
| NDN | + | 15 | 23930565 | 23932450 | 972 | 1885 | 0 | 0 | -0.052273904 | 0.331001753 | 0.330868612 |
| ATP10A | + | 15 | 25922420 | 26110317 | 4795 | 187897 | 1 | 5 | 0.501326386 | -0.175277912 | 0.074763692 |
| GABRB3 | + | 15 | 26788693 | 27184686 | 1772 | 395993 | 0 | 9 | 0.988016767 | -0.97958815 | -0.465274823 |
| GABRA5 | + | 15 | 27111510 | 27194354 | 1416 | 82844 | 0 | 6 | 0.802404149 | -0.548812563 | -0.157511534 |
| GABRG3 | + | 15 | 27216429 | 27778373 | 1428 | 561944 | 0 | 6 | 0.852648846 | -0.593942874 | -0.170777176 |
| OCA2 | + | 15 | 28000021 | 28344504 | 2714 | 344483 | 9 | 9 | -1.660380988 | -0.543931674 | -0.906499272 |
| APBA2 | + | 15 | 29129629 | 29410518 | 2647 | 280889 | 1 | 10 | 0.542886965 | -1.055651771 | -0.625635589 |
| NDNL2 | + | 15 | 29560353 | 29562033 | 921 | 1680 | 0 | 0 | 0.501962417 | 0.785228916 | 0.870913319 |
| TJP1 | + | 15 | 29991571 | 30261068 | 5441 | 269497 | 0 | 12 | 1.752442713 | -0.803995937 | -0.081921889 |
| MTMR10 | + | 15 | 31231144 | 31283810 | 2396 | 52666 | 14 | 28 | -2.293892804 | -2.030503686 | -1.928391813 |
| GRIN2A | + | 16 | 9852376 | 10276611 | 4549 | 424235 | 1 | 1 | 0.370715677 | 0.729710664 | 0.721094991 |
| MT1E | + | 16 | 56659387 | 56661024 | 492 | 1637 | 10 | 85 | -2.454760427 | -2.531252393 | -2.471627899 |
| MT1M | + | 16 | 56666145 | 56667898 | 315 | 1753 | 23 | 78 | -2.623544152 | -2.531252393 | -2.471627899 |
| MT1A | + | 16 | 56672578 | 56673999 | 204 | 1421 | 7 | 73 | -1.761405278 | -2.531252393 | -2.471627899 |
| MT1F | + | 16 | 56691606 | 56694610 | 245 | 3004 | 3 | 75 | -0.732291441 | -2.531252393 | -2.471627899 |
| CDH8 | + | 16 | 61681146 | 62070939 | 2528 | 389793 | 3 | 2 | -0.573570084 | 0.328517197 | -0.008246912 |
| SF3B3 | + | 16 | 70557691 | 70608820 | 3804 | 51129 | 0 | 2 | 1.529650601 | 0.972523491 | 1.375089727 |
| ZNF276 | + | 16 | 89786808 | 89807311 | 2703 | 20503 | 11 | 15 | -2.623544152 | -1.847806105 | -2.06366487 |
| FANCA | + | 16 | 89803957 | 89883065 | 5500 | 79108 | 67 | 32 | -2.623544152 | -2.531252393 | -2.471627899 |
| SCO1 | + | 17 | 10583654 | 10601692 | 942 | 18038 | 1 | 8 | 0.084061561 | -1.125325984 | -0.799948209 |
| ADPRM | + | 17 | 10600911 | 10614550 | 1059 | 13639 | 2 | 11 | -0.406990988 | -1.67909556 | -1.353159743 |
| TMEM220 | + | 17 | 10602332 | 10633633 | 441 | 31301 | 3 | 13 | -0.850692716 | -1.910378895 | -1.640173244 |
| RAPGEFL1 | + | 17 | 38333263 | 38351908 | 1540 | 18645 | 2 | 17 | 0.264748813 | -1.793686773 | -1.216594689 |
| RECQL5 | + | 17 | 73622925 | 73663269 | 3188 | 40344 | 9 | 4 | -1.875607855 | 0.249072756 | -0.624359689 |
| LGALS3BP | + | 17 | 76967320 | 76976191 | 1932 | 8871 | 0 | 0 | 0.083204782 | 0.538583426 | 0.54880306 |
| EPG5 | + | 18 | 43427574 | 43547240 | 8017 | 119666 | 1 | 1 | 0.987628271 | 1.386597994 | 1.417300608 |
| ABCA7 | + | 19 | 1040102 | 1065571 | 6865 | 25469 | 33 | 12 | -2.623544152 | -0.850864983 | -2.33107997 |
| ZNF317 | + | 19 | 9251056 | 9274100 | 1758 | 23044 | 0 | 1 | 0.546852862 | 0.473517186 | 0.670057364 |
| TECR | + | 19 | 14627897 | 14676792 | 1328 | 48895 | 5 | 14 | -0.762158576 | -1.361274273 | -1.144196973 |
| ZNF790 | + | 19 | 37308330 | 37341689 | 1935 | 33359 | 8 | 3 | -1.236542756 | 0.955106418 | -0.035262877 |
| ZNF568 | + | 19 | 37407231 | 37489602 | 3501 | 82371 | 3 | 3 | -0.015287437 | 0.752761724 | 0.404110078 |
| APOC2 | + | 19 | 45449243 | 45452822 | 336 | 3579 | 0 | 2 | 0.552719577 | 0.182312534 | 0.423901026 |
| CLPTM1 | + | 19 | 45457842 | 45496599 | 2500 | 38757 | 2 | 5 | -0.002170243 | -0.219791442 | -0.136935675 |
| SHANK1 | + | 19 | 51165084 | 51222707 | 6323 | 57623 | 0 | 3 | 1.140663112 | 0.46950049 | 0.789092733 |
| ZNF417 | + | 19 | 58411664 | 58427978 | 1746 | 16314 | 0 | 6 | 1.088952526 | -0.308218506 | 0.076195049 |
| ZNF343 | + | 20 | 2462463 | 2505348 | 1824 | 42885 | 0 | 10 | 0.81624114 | -1.361324352 | -0.795199446 |
| SSTR4 | + | 20 | 23016057 | 23017314 | 1173 | 1257 | 0 | 0 | 0.405605223 | 0.623262855 | 0.729431166 |
| CDH22 | + | 20 | 44802372 | 44937137 | 2555 | 134765 | 5 | 0 | -1.578268618 | 0.88145143 | -0.26589405 |
|  |  |  |  |  |  |  |  |  |  |  |  |
| OR5A1 | - | 11 | 59210617 | 59211667 | 954 | 1050 | 0 | 3 | 2.00330315 | 0.889007708 | 1.398695608 |
| MRPS17 | - | 7 | 55954970 | 56022932 | 744 | 67962 | 0 | 3 | 1.863136567 | 1.131022766 | 1.4653264 |
| IFNA6 | - | 9 | 21349834 | 21351377 | 576 | 1543 | 1 | 4 | 1.588574969 | 0.766991953 | 1.142146273 |
| KRT222 | - | 17 | 38785049 | 38821393 | 2051 | 36344 | 0 | 5 | 1.373846734 | 0.016502355 | 0.537578278 |
| IFNA8 | - | 9 | 21409146 | 21410184 | 576 | 1038 | 1 | 1 | 1.259921833 | 1.402169953 | 1.50898953 |
| IFNA2 | - | 9 | 21384254 | 21385396 | 573 | 1142 | 2 | 3 | 1.131156808 | 1.057697756 | 1.164000809 |
| KRT1 | - | 12 | 53068520 | 53074191 | 961 | 5671 | 0 | 1 | 1.079983152 | 0.943547389 | 1.203262807 |
| NPR1 | - | 1 | 153651113 | 153666468 | 3418 | 15355 | 0 | 17 | 1.025095634 | -1.936764709 | -1.247399084 |
| DCAF8 | - | 1 | 160185505 | 160254920 | 5519 | 69415 | 0 | 1 | 0.957237464 | 0.852765373 | 1.108323503 |
| MC3R | - | 20 | 54823788 | 54824871 | 1089 | 1083 | 0 | 0 | 0.902668364 | 1.011532036 | 1.208983609 |
| ZMYM6 | - | 1 | 35447134 | 35497342 | 2449 | 50208 | 0 | 2 | 0.869513173 | 0.40436517 | 0.718198843 |
| OR51A7 | - | 11 | 4928600 | 4929538 | 945 | 938 | 0 | 4 | 0.804757994 | -0.335079833 | 0.064676549 |
| NDUFB8 | - | 10 | 102265385 | 102289638 | 1649 | 24253 | 0 | 3 | 0.754240813 | 0.086982104 | 0.392793167 |
| PSMA2 | - | 7 | 42948872 | 42971773 | 2050 | 22901 | 0 | 3 | 0.712238121 | -0.008386022 | 0.321602379 |
| APOC4 | - | 19 | 45445495 | 45452820 | 738 | 7325 | 0 | 4 | 0.704400162 | -0.22857705 | 0.115620434 |
| APOC4-APOC2 | - | 19 | 45445495 | 45452822 | 738 | 7327 | 0 | 4 | 0.704400145 | -0.228577243 | 0.11562026 |
| TBPL2 | - | 14 | 55880259 | 55923444 | 1170 | 43185 | 0 | 2 | 0.701161562 | 0.274519725 | 0.555543593 |
| RNASE11 | - | 14 | 21051051 | 21077954 | 1076 | 26903 | 0 | 2 | 0.698384565 | 0.192699302 | 0.488118713 |
| ITGB3 | - | 17 | 45331212 | 45421658 | 2815 | 90446 | 1 | 7 | 0.627821824 | -0.513338287 | -0.133480891 |
| GPR179 | - | 17 | 36481413 | 36499730 | 7170 | 18317 | 0 | 16 | 0.603751417 | -2.140538837 | -1.575368664 |
| ZNF829 | - | 19 | 37379026 | 37407193 | 1417 | 28167 | 2 | 1 | 0.591881786 | 1.521491092 | 1.195510211 |
| NDUFA7 | - | 19 | 8373167 | 8386263 | 514 | 13096 | 0 | 9 | 0.585504558 | -1.2918201 | -0.829600132 |
| CCDC15 | - | 11 | 124824017 | 124911385 | 2827 | 87368 | 0 | 1 | 0.582799287 | 0.530860254 | 0.723666949 |
| ZNF223 | - | 19 | 44529506 | 44591471 | 3615 | 61965 | 1 | 9 | 0.547366961 | -0.957878289 | -0.506746903 |
| SDHD | - | 11 | 111957497 | 111990353 | 504 | 32856 | 1 | 1 | 0.508725421 | 0.923249361 | 0.892855517 |
| SNURF | - | 15 | 25200133 | 25223729 | 999 | 23596 | 1 | 9 | 0.494625639 | -0.958452948 | -0.535054016 |
| TIMM10B | - | 11 | 6502677 | 6505909 | 285 | 3232 | 0 | 2 | 0.475098274 | 0.128493226 | 0.34415333 |
| FPR3 | - | 19 | 52298416 | 52329442 | 1068 | 31026 | 0 | 79 | 0.470221145 | -2.531252393 | -2.471627899 |
| UQCR11 | - | 19 | 1578338 | 1605444 | 979 | 27106 | 0 | 3 | 0.469648729 | -0.140174772 | 0.102481605 |
| TLR9 | - | 3 | 52255096 | 52273183 | 4095 | 18087 | 0 | 0 | 0.467351633 | 0.842277503 | 0.940676644 |
| PSTPIP2 | - | 18 | 43563502 | 43652238 | 1084 | 88736 | 1 | 4 | 0.461399435 | -0.010655736 | 0.200411894 |
| MDGA2 | - | 14 | 47308826 | 48144157 | 3166 | 835331 | 1 | 3 | 0.454860699 | 0.114755413 | 0.295758017 |
| FPR2 | - | 19 | 52255279 | 52273779 | 1062 | 18500 | 0 | 44 | 0.431212051 | -2.531252393 | -2.471627899 |
| PSMA1 | - | 11 | 14515329 | 14541890 | 1333 | 26561 | 1 | 4 | 0.39544599 | -0.090300325 | 0.123998029 |
| TMEM239 | - | 20 | 2795614 | 2798712 | 811 | 3098 | 0 | 8 | 0.339550937 | -1.36434384 | -0.965477352 |
| F11R | - | 1 | 160965001 | 160991138 | 960 | 26137 | 1 | 4 | 0.300400902 | -0.128137195 | 0.049469062 |
| DDX60L | - | 4 | 169277886 | 169458937 | 5232 | 181051 | 2 | 3 | 0.294031669 | 0.513298286 | 0.489947409 |
| FFAR2 | - | 19 | 35934809 | 35942669 | 999 | 7860 | 0 | 0 | 0.284150195 | 0.561513087 | 0.647392864 |
| C6ORF165 | - | 6 | 88117701 | 88174183 | 1965 | 56482 | 1 | 2 | 0.2777637 | 0.349712688 | 0.415185071 |
| PI4K2A | - | 10 | 99344131 | 99433667 | 1410 | 89536 | 1 | 1 | 0.271314355 | 0.711172925 | 0.66933108 |
| HOXC4 | - | 12 | 54410715 | 54449813 | 807 | 39098 | 0 | 0 | 0.262721096 | 0.632613766 | 0.703834732 |
| TM9SF1 | - | 14 | 24658349 | 24682679 | 2681 | 24330 | 2 | 3 | 0.245780253 | 0.420389432 | 0.414767658 |
| TAP2 | - | 6 | 32781544 | 32806599 | 2779 | 25055 | 1 | 9 | 0.221428691 | -1.148708643 | -0.784288693 |
| OR5A2 | - | 11 | 59189416 | 59190449 | 981 | 1033 | 0 | 3 | 0.20882175 | -0.491335651 | -0.252152641 |
| FOXA2 | - | 20 | 22561643 | 22566093 | 1402 | 4450 | 0 | 0 | 0.164701281 | 0.522494276 | 0.570225949 |
| MFRP | - | 11 | 119209652 | 119217368 | 2342 | 7716 | 1 | 13 | 0.155107232 | -1.759853362 | -1.303418727 |
| SOGA3 | - | 6 | 127759551 | 127840146 | 4995 | 80595 | 1 | 0 | 0.115177061 | 0.934397255 | 0.779176604 |
| ZNF668 | - | 16 | 31072164 | 31085641 | 1942 | 13477 | 0 | 4 | 0.110829178 | -0.750576 | -0.525767985 |
| NPAP1 | - | 15 | 24920541 | 24928593 | 3477 | 8052 | 0 | 0 | 0.046415865 | 0.167295388 | 0.241857986 |
| CELF6 | - | 15 | 72559087 | 72612287 | 985 | 53200 | 1 | 0 | 0.03683415 | 0.937300396 | 0.732061151 |
| MRPL30 | - | 2 | 99771461 | 99811761 | 469 | 40300 | 1 | 97 | 0.028964383 | -2.531252393 | -2.471627899 |
| IER3IP1 | - | 18 | 44661027 | 44702652 | 633 | 41625 | 1 | 2 | 0.025075437 | 0.166352983 | 0.180038148 |
| SLC5A3 | - | 21 | 35445870 | 35478559 | 2214 | 32689 | 0 | 2 | -0.008765236 | -0.363896839 | -0.220639291 |
| ZNF747 | - | 16 | 30537244 | 30546173 | 2688 | 8929 | 0 | 0 | -0.039583786 | 0.365128257 | 0.360116263 |
| AQP1 | - | 7 | 30893010 | 30963427 | 1720 | 70417 | 3 | 2 | -0.067720053 | 0.829709529 | 0.519393278 |
| ALG9 | - | 11 | 111652919 | 111742305 | 1799 | 89386 | 2 | 2 | -0.102760105 | 0.439840777 | 0.292094762 |
| ZFP41 | - | 8 | 144328991 | 144344875 | 603 | 15884 | 1 | 0 | -0.109732969 | 0.717745364 | 0.510519046 |
| MT1B | - | 16 | 56685811 | 56687116 | 231 | 1305 | 2 | 74 | -0.110008246 | -2.531252393 | -2.471627899 |
| CRIP1 | - | 14 | 105952654 | 105955284 | 212 | 2630 | 1 | 2 | -0.136071241 | 0.091043029 | 0.045688945 |
| SYCP2 | - | 20 | 58438618 | 58508710 | 4933 | 70092 | 4 | 9 | -0.141331208 | -0.473549305 | -0.316503603 |
| CHMP4C | - | 8 | 82644669 | 82671750 | 732 | 27081 | 1 | 3 | -0.1488854 | -0.230913621 | -0.192377797 |
| DENND2C | - | 1 | 115125469 | 115213043 | 2721 | 87574 | 3 | 1 | -0.166017758 | 1.016912902 | 0.613251714 |
| EFNA3 | - | 1 | 155036224 | 155059283 | 732 | 23059 | 1 | 0 | -0.284459084 | 0.686259544 | 0.412188207 |
| SLC10A5 | - | 8 | 82605842 | 82608409 | 1323 | 2567 | 2 | 2 | -0.291947566 | 0.104626332 | -0.066575942 |
| ZNF709 | - | 19 | 12571998 | 12624668 | 1950 | 52670 | 2 | 2 | -0.347885862 | 0.165404134 | -0.004170961 |
| IFNA13 | - | 9 | 21367371 | 21368075 | 577 | 704 | 1 | 4 | -0.375814849 | -0.724692337 | -0.663193596 |
| LTB4R2 | - | 14 | 24774940 | 24781259 | 1315 | 6319 | 1 | 0 | -0.384529414 | 0.51856652 | 0.242996017 |
| SOX7 | - | 8 | 10581278 | 10697357 | 1618 | 116079 | 1 | 9 | -0.385358363 | -1.61821428 | -1.380663381 |
| LSP1 | - | 11 | 1874200 | 1913497 | 938 | 39297 | 2 | 3 | -0.431311613 | -0.049404415 | -0.199708948 |
| ZNF8 | - | 19 | 58790317 | 58807254 | 1680 | 16937 | 2 | 3 | -0.499181617 | -0.199688553 | -0.334719242 |
| ASGR2 | - | 17 | 7004641 | 7019019 | 906 | 14378 | 3 | 4 | -0.606254293 | -0.156681805 | -0.333131143 |
| ACE | - | 17 | 61554422 | 61599205 | 4273 | 44783 | 4 | 2 | -0.630531362 | 0.711424805 | 0.190219613 |
| CCDC178 | - | 18 | 30517366 | 31021065 | 2730 | 503699 | 4 | 1 | -0.639756324 | 0.86839966 | 0.260166627 |
| SLC25A10 | - | 17 | 79670401 | 79687569 | 1404 | 17168 | 4 | 5 | -0.692123393 | -0.152211722 | -0.351092297 |
| C2ORF15 | - | 2 | 99757948 | 99767950 | 390 | 10002 | 3 | 86 | -0.751355563 | -2.531252393 | -2.471627899 |
| LIPN | - | 10 | 90521163 | 90537999 | 1224 | 16836 | 3 | 3 | -0.80807055 | -0.090253703 | -0.374849419 |
| PIK3R2 | - | 19 | 18263928 | 18281350 | 1678 | 17422 | 3 | 0 | -0.818207213 | 0.966641614 | 0.234346542 |
| MYH3 | - | 17 | 10531843 | 10560626 | 6057 | 28783 | 8 | 9 | -0.83613782 | -0.059029141 | -0.227385648 |
| CFB | - | 6 | 31895475 | 31919825 | 4797 | 24350 | 8 | 3 | -1.21655474 | 0.860292159 | 0.019899919 |
| OC90 | - | 8 | 133036467 | 133071627 | 1424 | 35160 | 4 | 4 | -1.22297483 | -0.318979538 | -0.70733283 |
| UGT2A1 | - | 4 | 70454135 | 70518965 | 1620 | 64830 | 5 | 8 | -1.452397597 | -1.157114238 | -1.290499802 |
| CDRT1 | - | 17 | 15468797 | 15469590 | 735 | 793 | 5 | 13 | -1.583552773 | -1.987581379 | -1.933008812 |
| ZNF763 | - | 19 | 12035890 | 12090105 | 3340 | 54215 | 7 | 13 | -1.672130671 | -1.613127348 | -1.620374567 |
| PCDH15 | - | 10 | 55562531 | 57387702 | 7759 | 1825171 | 15 | 5 | -1.70907171 | 0.649013663 | -0.270961846 |
| TMBIM4 | - | 12 | 66517697 | 66563765 | 1009 | 46068 | 6 | 7 | -1.713035924 | -0.935854517 | -1.24193792 |
| NAA60 | - | 16 | 3415099 | 3536960 | 1755 | 121861 | 5 | 3 | -1.7214776 | -0.241712945 | -0.899431715 |
| KLK9 | - | 19 | 51499274 | 51512837 | 1530 | 13563 | 7 | 2 | -1.737563801 | 0.543418516 | -0.486487867 |
| TM4SF19 | - | 3 | 196042953 | 196065244 | 1067 | 22291 | 6 | 8 | -1.74542378 | -1.052405841 | -1.353431098 |
| ITFG3 | - | 16 | 284545 | 319942 | 2047 | 35397 | 8 | 17 | -2.119119744 | -2.137781451 | -2.083388631 |
| MYH1 | - | 17 | 10395624 | 10421860 | 6048 | 26236 | 14 | 66 | -2.145800473 | -2.531252393 | -2.471627899 |
| KIAA0391 | - | 14 | 35591052 | 35743271 | 1858 | 152219 | 8 | 17 | -2.363937756 | -2.388528159 | -2.349853415 |
| PGBD2 | - | 1 | 249200395 | 249214145 | 1791 | 13750 | 10 | 44 | -2.372034031 | -2.531252393 | -2.471627899 |
| F2RL2 | - | 5 | 75911307 | 75919259 | 1137 | 7952 | 9 | 1 | -2.394665006 | 0.582109157 | -0.860471779 |
| MS4A10 | - | 11 | 60552821 | 60568778 | 758 | 15957 | 11 | 2 | -2.623544152 | 0.341150501 | -1.151351654 |
| MS4A15 | - | 11 | 60524426 | 60544205 | 627 | 19779 | 11 | 2 | -2.623544152 | 0.386350104 | -1.100406599 |
| FAM170A | - | 5 | 118965254 | 118971517 | 1064 | 6263 | 12 | 2 | -2.623544152 | 0.470445528 | -1.080167359 |
| OR51T1 | - | 11 | 4903049 | 4904113 | 1071 | 1064 | 12 | 3 | -2.623544152 | -0.312879774 | -1.741515134 |
| GALT | - | 9 | 34638130 | 34651032 | 1279 | 12902 | 18 | 6 | -2.623544152 | -0.256425303 | -1.622835199 |
| PAGR1 | - | 16 | 29827285 | 29841948 | 914 | 14663 | 19 | 28 | -2.623544152 | -2.218653397 | -2.356307677 |
| MYH2 | - | 17 | 10424465 | 10453274 | 6112 | 28809 | 20 | 20 | -2.623544152 | -1.479661087 | -1.744933332 |
| LCN6 | - | 9 | 139632619 | 139642905 | 1113 | 10286 | 21 | 7 | -2.623544152 | -1.050552283 | -2.471627899 |
| MUSK | - | 9 | 113431051 | 113563859 | 2867 | 132808 | 24 | 10 | -2.623544152 | -0.962658632 | -2.194517064 |
| LRRC37A3 | - | 17 | 62850430 | 62915598 | 4656 | 65168 | 29 | 32 | -2.623544152 | -2.531252393 | -2.471627899 |
| SHPK | - | 17 | 3468738 | 3539543 | 4111 | 70805 | 67 | 14 | -2.623544152 | -1.803423642 | -2.471627899 |
| TRPM1 | - | 15 | 31293264 | 31453476 | 5046 | 160212 | 68 | 32 | -2.623544152 | -1.725519217 | -2.471627899 |
| CAPN11 | - | 6 | 44126548 | 44152139 | 2274 | 25591 | 88 | 106 | -2.623544152 | -2.531252393 | -2.471627899 |
| KRT77 | - | 12 | 53083410 | 53097247 | 1510 | 13837 | 165 | 3 | -2.623544152 | 0.387708152 | -2.471627899 |
| AOC1 | - | 7 | 150521715 | 150558592 | 2336 | 36877 | 251 | 1 | -2.623544152 | 0.502388617 | -2.471627899 |

Data is sorted from low to high deletion score (del.score) and duplication (dup) frequencies. "+" indicates expression in the brain. Deletion score increases with increasing intolerance.
